# Detecting and Monitoring Hate Speech in Twitter

**DOI:** 10.3390/s19214654

**Published:** 2019-10-26

**Authors:** Juan Carlos Pereira-Kohatsu, Lara Quijano-Sánchez, Federico Liberatore, Miguel Camacho-Collados

**Affiliations:** 1 Engineering School, Autonomous University of Madrid, 28049 Madrid, Spain; jc.pereira.kohatsu@gmail.com; 2 UC3M-BS Institute of Financial Big Data, Charles III University of Madrid, 28903 Madrid, Spain; 3 School of Computer Science & Informatics, Cardiff University, Cardiff CF24 3AA, UK; LiberatoreF@cardiff.ac.uk; 4 Artificial Intelligence Area Coordinator, Cabinet of the Secretary of State for Digital Advancement, 28020 Madrid, Spain; mcamachoc@mineco.es; 5 State Secretariat for Security, Interior Ministry, 28010 Madrid, Spain

**Keywords:** hate crime, sentiment analysis, text classification, predictive policing, social network analysis, Twitter

## Abstract

Social Media are sensors in the real world that can be used to measure the pulse of societies. However, the massive and unfiltered feed of messages posted in social media is a phenomenon that nowadays raises social alarms, especially when these messages contain hate speech targeted to a specific individual or group. In this context, governments and non-governmental organizations (NGOs) are concerned about the possible negative impact that these messages can have on individuals or on the society. In this paper, we present *HaterNet*, an intelligent system currently being used by the Spanish National Office Against Hate Crimes of the Spanish State Secretariat for Security that identifies and monitors the evolution of hate speech in Twitter. The contributions of this research are many-fold: (1) It introduces the first intelligent system that monitors and visualizes, using social network analysis techniques, hate speech in Social Media. (2) It introduces a novel public dataset on hate speech in Spanish consisting of 6000 expert-labeled tweets. (3) It compares several classification approaches based on different document representation strategies and text classification models. (4) The best approach consists of a combination of a LTSM+MLP neural network that takes as input the tweet’s word, emoji, and expression tokens’ embeddings enriched by the tf-idf, and obtains an area under the curve (AUC) of 0.828 on our dataset, outperforming previous methods presented in the literature.

## 1. Introduction

Worldwide accessibility to the Internet has incredibly reshaped our perception of the world. One of the children of the World Wide Web is Social Media (SM), which is present in many forms: online game platforms, dating apps, forums, online news services, and social networks. Different social networks aim at different objectives: opinion transmission (Twitter or Facebook), business contacts (LinkedIn), image sharing (Instagram), video transmission (YouTube), dating (Meetic), and so on. However, they all have one thing in common: they aim to connect people. The power of social networking is so great that the number of worldwide users is expected to reach 3.02 billion active social media users per month by 2021. This will account for approximately one-third of the Earth’s population.

Among the many existing social networks, Twitter currently ranks as one of the leading platforms and is one of the most important data sources for researchers. Twitter is a well-known real-time public microblogging network where, frequently, news appear before than on official news media. Characterized by its short message limit (now 280 characters) and unfiltered feed, its usage has quickly escalated, especially amid events, with an average of 500 million tweets posted per day.

In recent years, social networks (and especially Twitter) have been used to spread hate messages. Hate speech refers to a kind of speech that denigrates a person or multiple persons based on their membership to a group, usually defined by race, ethnicity, sexual orientation, gender identity, disability, religion, political affiliation, or views. The Rabat Plan of Action of the United Nations [1], which defines the guidelines to distinguish between free speech and hate speech, recommends differentiating between three types of expressions: “expression that constitutes a criminal offence; expression that is not criminally punishable, but may justify a civil suit or administrative sanctions; expression that does not give rise to criminal, civil or administrative sanctions, but still raises concern in terms of tolerance, civility and respect for the rights of others.”

Related to this, hate crimes are a type of violation of the law whose primary motivation is the existence of prejudices regarding the victims. This occurs when the offender chooses victims on grounds that they belong to a certain group defined basically by the attributes mentioned earlier. There is evidence that hate crimes are influenced by singular widely publicized events [2] (terrorist attacks, uncontrolled migration, demonstrations, riots, etc.). These events usually act as triggers, and their effect is dramatically increased inside SM. This makes SM a sensor in the real world [3] and a source of valuable information for crime forecasting [4]. In fact, social networks are filled with messages from individuals inciting punishment against different targeted groups. When these messages are collected after a trigger event over a period of time, they can be used for the analysis of hate crimes in all the phases [2]: climbing, stabilization, duration, and decline of the threat. Therefore, monitoring SM becomes a priority for the forecasting, detection and analysis of hate crimes.

Following this necessity, the main goal of this paper is the design of a system, *HaterNet*, capable of identifying and classifying hate speech in Twitter, as well as monitoring and analyzing hate trends and other negative sentiments. This system allows to detect triggers of hate waves, especially against minority groups and individuals belonging to these groups. This provides valuable information for security agencies and police corps, especially to predict future crimes or to take measures afterwards. In fact, *HaterNet* has been developed in collaboration with the Spanish State Secretariat for Security of the Ministry of Interior (SES) and, more concretely, the Spanish National Office Against Hate Crimes (SNOAHC-SES). However, the methodology described in this paper is not country- or language-specific and could be localized. Due to the application context, the system presented in this research studies exclusively the first two types of expressions identified by the Rabat Plan of Action (as they can give rise to suits or sanctions). The study of the third one, which is more subjective in nature, is left as future work.

*HaterNet* is comprised of two modules:

1. Hate Speech Detection: tweet collection and classification
2. Social Network Analyzer

The first module deals with sampling and classifying tweets, whereas the second one provides tools to analyze the hate speech content inside the social network.

As further detailed next, the contributions related to hate speech classification research are mainly twofold. (1) A new public dataset that can be used to test, train, and benchmark new developed methods. This contribution is very relevant as authors have so far only found three public hate-speech datasets [5,6,7]. The first two datasets have the inconvenience of consisting of ids of tweets to download. As Twitter periodically erases some tweets, especially if offensive, it is currently not possible to download the original datasets presented in [5,6]. Besides, most of the related literature on hate speech is benchmarked against them [8,9,10,11]. Therefore, it is crucial for future advances in this domain to provide new self-contained public datasets. (2) A new classification approach, referred as a double deep learning neural approach, consisting of a combination of a Long Short-Term Memory (LTSM) and a Multilayer Perceptron (MLP) neural network that takes as input words, emojis, and expression tokens’ embeddings of a tweet, enriched by the tf-idf. The embeddings are, in turn, obtained using a neural network-based word2vec methodology. Our experiments have shown that this approach outperforms reference models in the literature.

It is in the second module, the “Social Network Analyzer”, where the main novelty of this paper lies. In fact, the literature on hate speech so far has been concerned only on studying and proposing methods that classify texts as containers of hate speech or not. We go one step further: the classification provided by *HaterNet* can be used to build networks of concepts and actors, according to their relationship to the hate messages, which can then be visually represented. To the best of the authors’ knowledge, this is the first system with these characteristics that has been presented in the literature [12]. There are many services and application contexts that can benefit from it. For instance, it provides an interpretable graphical representation of the classification algorithm functioning. Whereas other systems are usually black-boxes, *HaterNet* illustrates the relevant terms, receivers, and emitters identified inside hate speech texts. This feature could be used for either further studying correlations or different aspects inside hate containing text, or for detecting biases. Transparency and explainability are currently hot topics in the area of artificial intelligence tools [13], as well as bias in classification algorithms [14]. Another usage of this function is related to policing and journalism. In fact, observers can use *HaterNet* to analyze the evolution of different hate trends, key words, or actors; study their triggers; and design protocols to mitigate concrete peaks of hate; among others.

Furthermore, this research spans two scopes and provides methodological and applied contributions: (1) in text classification, and (2) in the design, development, testing, and implementation of an artificial intelligence system capable of recognizing, interpreting, processing, and visualizing the state of a specific sentiment (i.e., hate). With regards to the latter the contributions are as below.

- The design and implementation of a novel intelligent system for monitoring hate speech in SM. In particular, *HaterNet* acts as a visual thermometer of emotions that allows to map the hate state of a region and its evolution by targeting, aspects, emitters, and receivers of hate.
- A novel addition to the literature on predictive policing, as *HaterNet* has been developed to carry out policing tasks. Note that it could be used in other investigation fields, such as journalism or social analysis.
- The definition of a methodology that combines text classification and social network analysis to monitor the evolution of classes of documents (in this case, hate speech) in SM and identify the main actors and groups involved.

With respect to the text classification area, the contributions are as below.

- The introduction of a new approach based on double deep learning neural networks.
- A novel public dataset in Spanish on hate speech, comprised of two million untagged tweets and 6000 tagged tweets.
- A thorough comparison of text classification methodologies on datasets from the literature and on a new real-world corpus, where the results show that the model proposed in this paper provides better performance than previous models in all the datasets considered.
- A study on the significance of including suffixes in text classification models. To the best of the authors knowledge, this is the first model to explicitly consider suffixes in the Spanish language.

The remainder of this paper is structured as follows. In the next section, we introduce some of the state-of-the-art research regarding predictive policing, SM predictive systems, sentiment analysis, and hate speech detection. Section 3 illustrates the theoretical concepts and design of the text classification module. Implementation details are given in Section 4. The computational experiments undertaken on the classification models are the subject of Section 5. Following, Section 6 analyzes the obtained results and compares them against the state-of-the-art literature allowing for a discussion and providing some insights. Next, Section 7 presents the user interface tools of the Social Network Analyzer module and the applications of *HaterNet* for the Spanish government. Section 8 summarizes the architecture and functioning of *HaterNet*. Finally, Section 9 concludes the paper and proposes future lines of research.

## 2. Related Work

As the volume of papers published in the area of SM proves, analyzing, understanding, and studying social networks are a major research challenge [15]. Currently, one of the main concerns about SM usage is the impact (either positive or negative) that specific messages can have on individuals or on the society. This is the reason why new research topics like *Social Network Analysis* and *Sentiment Analysis* have become important fields of study and interest not only for academics, but also for companies and governments.

Sentiment analysis is a family of techniques that is widely used in areas such as marketing, social networking development, reviews, survey responses, and especially in customer service [16]. It uses data science methods like *Natural Language Processing* (NLP), *Machine Learning* (ML), and *Statistics* to determine how an individual or group of people feels and reacts to a specific situation or comment [17]. It can be also used to perform geolocation analysis to determine political feelings (e.g., which political leader has better acceptance), marketing preferences (e.g., competitor’s products opinion over population), or, in general, any statistical analysis on text data [18].

Through sentiment analysis, and more concretely ML classification algorithms, messages in a social network such as Twitter can be analyzed and tagged to determine the writer’s attitude on a topic. In our case, we design and implement a sentiment analysis model to identify tweets that contain hate speech.

In the following subsections, a brief summary of previous work in the context of predictive policing, SM used as a predictive tool, sentiment analysis, and hate speech detection is given.

### 2.1. State-of-the-Art on Predictive Policing

Predictive policing consists of the use of quantitative analysis methods to detect and forecast potential criminal activity. To date, there are four different types of predictive policing strategies [19]:

- Methods for predicting felonies: used to forecast places and times with crime escalation.
- Methods for predicting transgressors: used to identify individuals at risk of committing a felony in the future.
- Methods for predicting transgressors’ identities: used to shape profiles that precisely match likely transgressors with specific past felonies.
- Methods for predicting victims of felonies: used to identify groups, prototypes, or, in some cases, individuals who are likely to become victims of a felony.

The system here proposed, *HaterNet*, belongs to the last group. Also, it could be part of a system that predicts future felonies (hate crimes in particular), transgressors’ profiles, and identities. In fact, by detecting hate speech, analyzing the targets, and the spatiotemporal characteristics, actions can be taken to identify possible victims and transgressors.

Examples of other works that support police actions through modeling are given in the following. Cohen et al. [20] predicted violence in crimes with an accuracy of $R^{2}=\left[0.69-0.79\right]$ by using a linear regression based model aided by neural networks. Yu et al. [21] forecasted crime hotspots through the usage of ML classification techniques on crime records. On the other hand, Kang and Kang [22] predicted crime occurrence using a deep neural network (DNN), on a dataset collected from various online databases, obtaining an area under the curve (AUC) of 0.833. Recently, Quijano-Sánchez et al. [23] developed *VeriPol*, a system currently in use by the Spanish National Police, to infer the probability of falsehood of police reports. *VeriPol* combines NLP and ML to achieve a success rate of more than 91%.

Related to monitoring crime and improving patrolling, hot-spot maps are a traditional method for analyzing and visualizing the distribution of crimes across space and time [24,25]. Future crimes often occur in the vicinity of past crimes, making hot-spot maps a valuable prediction tool. More advanced techniques use time series [26] and self-exciting point process models to capture the spatiotemporal clustering of criminal events [27]. These techniques, as pointed out in Bendler et al. [28], are useful but carry specific limitations, as they do not consider the rich SM landscape when analyzing crime patterns.

### 2.2. State-of-the-Art on Twitter Data for Predictive Analytics

With the increasing expansion of social media networks, our capacity to interact, collaborate, and network has highly and rapidly increased [29]. In this context, research in a number of academic fields that involve both the transformation of noisy raw data and the design of analysis methods has shown the incredible predictive power of SM, and more concretely Twitter, to forecast political reactions [30] or disease outbreaks [31]. In their survey paper Kalampokis et al. [32], the authors identify seven application areas: disease outbreaks [33], election results [17], macroeconomic processes [34], box office performance of movies [35], natural phenomena such as earthquakes [36], product sales [37], and financial markets [38]. Among the 52 articles reviewed, only Wang et al. [34] applied SM analysis to crime prediction. More recently, Gerber [39] presented a linear regression strategy to predict local criminal activity in Chicago that combines standard Kernel Density Estimation (KDE) with a topic modeling algorithm trained on Twitter content. Using a similar modeling strategy, Chen et al. [40] improved on the above-mentioned work by adding weather features and the polarity of the revised Twitter data, estimated through a sentiment lexicon dictionary.

### 2.3. State-of-the-Art on Text Classification

Sentiment analysis is a text classification technique used to discern opinions and sentiments in text [41]. To date, there are three different approaches to complete this task:

- Lexicon-based [40,42], which uses a predefined lexicon to check the occurrence of words in the revised text.
- ML-based [39,43], which uses language model classifiers, being linear regression the most common [39].
- Deep learning techniques [43], which learn complex features using deep neural networks.

The first two approaches are known as surface forms, as they use lexical and syntactical information to search for text patterns, whereas the third approach relies on semantic aspects. Surface forms consider that each document is represented as a set of terms and their frequency in the document. These models make use of approaches, such as *Bag Of Words* (BOW), *n-grams*, or *Part-of-Speech tagging* (POS), and have worked remarkably well for many NLP problems [23,44]. However, they are not capable of explaining the word semantics. Methods based on word embeddings make possible to come close to this objective. Their goal is to represent terms in a high dimensional space, while preserving their semantic relationships. There are different models that create word embeddings, word2vec [45] being the most popular. This model generates embeddings by training a neural network with a single hidden layer. It is necessary to set the size of the neighborhood of a word, i.e., the other words that surround it and that define its context. The training process consists of trying to predict the neighbors of each input word. Word2vec uses self-supervised learning, and its rationale is that the semantics of a term depend on the neighbors of such term. The length of the generated word embedding depends on the number of neurons in the hidden layer.

With respect to sentiment analysis, Kalampokis et al. [32] drew the following conclusions.

- So far, prediction results in sentiment analysis have been poor due to the informal and noisy nature of SM, which creates problems for NLP tools [42]. However, as illustrated in Section 5.3, *HaterNet* obtains an AUC of 0.828, which denotes a reliable classification.
- Studies in this area are mainly focused on stock market and leisure reviews [38,43,46,47,48,49].
- The use of non-SM predictor variables is mainly limited to past values of objective phenomenons (weather and budgets) and demographics [35,40]. In contrast, *HaterNet* uses prediction features (e.g., the appearance of suffixes) extracted from the text by applying NLP techniques.

More recently, Quijano-Sánchez et al. [23] presented a text classification approach that discerned between truthful and deceptive texts. The proposed algorithm was tested against the following state-of-the-art sentiment analysis approaches and proved more accurate across a wide number of evaluation metrics (refer to Section 3.6):

- Ott et al. [46,47], Hernández Fusilier et al. [48], and Cagnina and Rosso [49] addressed text classification between positive and negative sentiment in hotel opinions.
- Mihalcea and Strapparava [50] tackled text classification between truthful and false abortion, death penalty, and friends opinions.
- Li et al. [51] focused on text classification between positive and negative sentiment in hotels, restaurants, and doctors opinions.

Thus, in this paper, the approach followed by Quijano-Sánchez et al. [23] is taken as gold standard. Our research improves on it by considering more document representations and more complex models.

Work Araque et al. [43] comes closest to the present research by using ensemble techniques that combine both deep learning approaches and traditional surface methods. However, although their base classifier consists of a word embedding model and a linear ML algorithm, our double deep learning approach consists of a word embedding model and a neural network ML algorithm combined with frequency features. Araque et al. [43] also present several ensemble classifiers, combining their baseline with state-of-the-art surface classifiers, that outperform each of these methodologies individually. However, as explained in Section 6, our double deep learning approach surpasses the methodologies proposed by Araque et al. [43] and, therefore, all the other approaches they tested themselves against.

### 2.4. State-of-the-Art on Hate Speech Detection

Recently, there has been a growing interest in the application of text classification models for the detection of hate speech, especially in the context of online outlets, such as social media and web blogs. This trend has resulted in a number of papers published on the subject in the last years. Most researches focused on applying text classification models to datasets obtained from online sources, with different degrees of success [12].

Table 1 presents a summary of the main characteristics of each contribution in the literature. In particular, the features adopted, the training models studied, the datasets employed in the experiments, their availability, and the reported performances are summarized. Djuric et al. [52] extracted approximately 950k Yahoo Finance user comments and applied paragraph2vec for joint modeling of whole comments and words, using the continuous BOW (CBOW) neural language model. The embeddings are then used in a LR model to classify the comments into hate or clean. Zia et al. [53] focused on the problem of identifying hatred in the context of spiritual belief (i.e., Christianity, Islam, and Judaism) based on Twitter dataset. Unfortunately, the size of the dataset is not specified in the paper. The authors compared different classical machine learning models and found that Support Vector Machine (SVM) performs the best. Silva et al. [54] used sentence structure rules and a hate thesaurus to identify hate in whispers and tweets. Despite the humongous size of their dataset (more than 27M whispers and 512M tweets), only 100 messages identified as hateful by their model have been used to validate it. Waseem and Hovy [5] provided the first of two widely used datasets. In particular, the dataset is comprised of 16,914 annotated tweets, of which 3383 are categorized as “sexist” and 1972 as “racist”. The corpus was annotated by the two authors and a student in gender studies to mitigate the bias, showing an inter-annotator agreement of κ=0.84. In terms of model, the authors consider gender, length, location, character, and word n-grams features, which are used to estimate a LR classification model. The second dataset, comprised of 6909 annotated tweets, was introduced in Waseem [6]. It includes 3000 tweets from the previous dataset, albeit with new annotations, and 4000 new tweets. The annotation is carried out by two groups of users: experts (mostly feminist and anti-racism activist) and amateurs (600 crowd-sourced labelers). It is found that the inter-annotator agreement among the amateur annotators is κ=0.57(σ=0.08). The authors stated that the low value is an evidence in favor of the claim by Ross et al. [55] that stated that the annotation of hate speech is a hard task. With respect to the classification model, the authors expanded on their previous contribution by adding skip-gram, cluster, POS, and Author Historical Salient Terms features, significantly improving their LR classification model. Badjatiya et al. [11] (Code available at https://github.com/pinkeshbadjatiya/twitter-hatespeech) used the 16,000 tweet dataset by [5]. The authors tested different configurations of features and models (see Table 1). The best results, which outperform those of [5,10], are obtained by using random word embeddings, LSTM, and Gradient Boosted Decision Trees (GBDT). Davidson et al. [7] (Code available at https://github.com/t-davidson/hate-speech-and-offensive-language) introduced a novel dataset comprised of almost 25,000 tweets labeled using crowdsourcing. The authors characterized the messages through features based on words and other aspects of the tweet, such as readability, sentiment, hastags, mentions, retweets, and URLs. These features were used to train classical machine learning models (i.e., Logistic Regression (LR), Naïve Bayes (NB), Decision Trees (DT), Random Forests (RF), and linear SVMs). The authors, that found that LR and Linear SVM performed significantly better than other models, and chose a LR model with L2 penalization due to explainability reasons. Gambäck and Sikdar [8] applied CNNs to a subset of 6655 tweets from dataset [6], as some of the annotated tweets were unavailable or had been deleted from Twitter because of its offensive content. The authors tested the classification capabilities of word embeddings and character n-grams. The results obtained, though inferior to those of Waseem [6], are not fully comparable due to the differences in the dataset. Park and Fung [9] merged the dataset in [5], with the tweets annotated by experts in [6], in a single dataset. The authors explored the impact of using character and word embeddings in three different CNN architectures. Del Vigna et al. [56] combined morpho-syntactical features, sentiment polarity, and word embedding lexicons to classify a novel dataset of 6502 annotated Facebook comments in Italian, using SVM and LSTM. The reported inter-annotator agreement on this dataset is of κ=0.26, stressing again the difficulty of annotating hate speech. Also, Salminen et al. [57] introduced a new dataset. The authors annotated 5143 comments from Twitter and Facebook videos, and tested word embeddings, and semantic and syntactic features on classical Machine Learning algorithms. The best performance was obtained using linear SVM. Unfortunately, the two datasets are not publicly available. Finally, Zhang et al. [10] (Code available at https://github.com/ziqizhang/chase) tackled the problem of classifying hateful comments in five datasets: [5,6], the combination of [5,6] used in [9]; the 25k labeled tweets from  Davidson et al. [7]; and a novel dataset of 2435 annotated tweets. In terms of features, the authors expanded on those adopted in the previous papers by adding mentions, misspellings, emojis, punctuation, and capitalization features. The tested models were SVM and CNNs combined with GRU (Gated Recurrent Unit Networks), providing better results the latter. In terms of performance, reported results show an improvement on the approaches described in [5,6,7]. However, they are inferior to the models proposed by Badjatiya et al. [11] and Park and Fung [9].

## 3. Hate Speech Detection: Design and Theoretical Concepts

**Figure 1:**
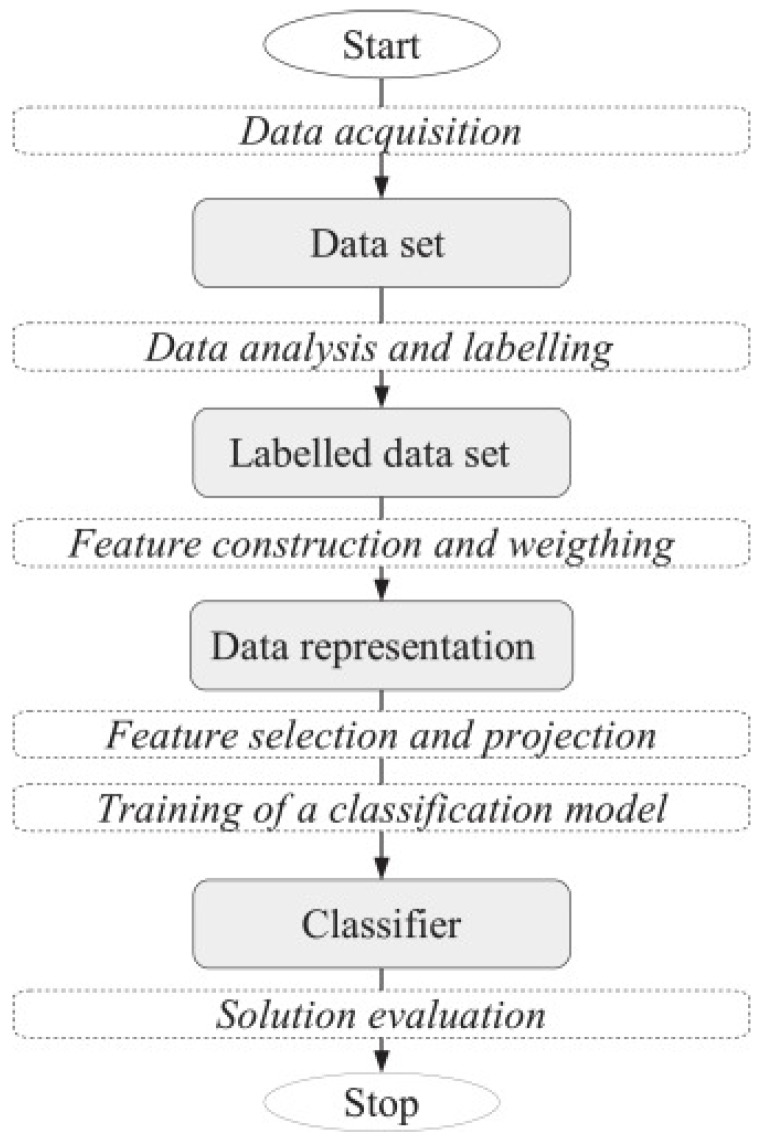
Standard text classification process (source: Mirończuk and Protasiewicz [58]).

*HaterNet*’s first module, *Hate Speech Detection*, periodically collects tweets and classifies them as containers of hate speech or not. This module is comprised of the following steps, conceptualized in Figure 1. We refer the reader to the survey by Mirończuk and Protasiewicz [58] for a comprehensive overview of the essential phases of text classification.

### 3.1. Corpus Collection and Cleaning

*HaterNet*’s classification process uses a supervised learning approach where an ML function is inferred from labeled training data. An initial corpus of two million tweets is downloaded using Twitter’s API Rest. This “raw” corpus is the base of the model’s training dataset. Next, a cleaning step prepares the texts for processing, i.e., dealing with capitalization, uncommon characters, symbols, etc.

### 3.2. Document Selection

The number of tweets containing hate speech in a given period of time is very low, usually less than 1%. This fact poses two difficulties that must be addressed: (1) imbalanced classes and (2) manually tagging a relevant dataset. Thus, to obtain a representative and balanced training dataset that can be tagged by experts, it is necessary to previously filter the initial two million downloaded tweets. This necessity is even more acute in our specific application as the training dataset needs to be systematically renewed, and therefore it has to be continuously tagged, due to the ongoing evolution of trends and hate targets in social media. Note that, at the end of this stage, the training set becomes more balanced than the raw corpus.

### 3.3. Document Labeling

The filtered tweets are manually classified to produce both training and test sets. In *HaterNet*, this task is executed by four different raters. Authors are aware of the possible bias of having a limited and nonrandomized set of experts tagging the sample and leave for future work the development of a nonbiased corpus.

### 3.4. Document Representation and Feature Extraction

Prior to classifying, tweets are processed to generate representative features that can be then provided as inputs to the classification models. Initially, an NLP preprocessing pipeline [58] is followed: lowercasing, tokenization, POS tagging, lemmatization, and stopword removal for the obtention of unigrams. The software TreeTagger [59] is used for tokenization, lemmatization, and POS tagging. Although tokenization and stopword removal are standard prior steps for normalizing texts, lower-casing and lemmatization are aimed at reducing sparsity and vocabulary size, steps which have been proved beneficial in text classification tasks. As far as the stopword removal step is concerned, in our system, a word is considered as stopword if it is not a verb, a noun, an adjective, or an adverb. For a list of the POS tags considered and their description, please refer to [60].

A new feature representing word suffixes is included. Suffixes in Spanish tend to be useful in tweet representation because they add different inflections and meanings to a word. As an example, the suffix “-ucho” is used for obtaining new nouns and adjectives that are usually derogatory: the word “hotel” has the same meaning as in English, whereas “hotelucho” means “fleabag hotel”. Obviously, given *HaterNet*’s classification goal, the detection of pejorative aspects could be relevant. Finally, new tokens are created to unify different nonverbal expressions (e.g., URLs, hashtags, and marks).

After this processing, two types of features are extracted:

- Frequency-based: Computed for unigrams, POS tags, emojis, suffixes, and expression tokens. All the following types of frequencies are considered for each feature.
  - Absolute frequency, $f_{t,d}$: the number of times that term *t* occurs in document *d*.
  - Binary transformation, $f_{t,d}^{b}$: equals 1 if $f_{t,d}>0$, and 0 otherwise.
  - Logarithm transformation, $\log(1+f_{t,d})$.
  - Ratio transformation, $\frac{f_{t,d}}{\sum_{t^{\prime }\in d}f_{t^{\prime },d}}$. Adjusts the absolute frequency to the document length.
- Embeddings-based: Words, emojis, suffixes, and tokens embeddings are obtained using word2vec. These embeddings can be extended by attaching them POS tags (transformed using one-hot encoding) and Text Frequency-Inverse Document Frequency (tf-idf) Kusner et al. [61] information. Tf-idf represents the importance of a term in a document relative to the whole corpus. It is based on the idea that a term that appears many times inside a document must be relevant for that document, but if it appears many times in other documents, its relevance decreases.

Also, both representations can be complemented by adding the number of words, lemmata (total and unique), and sentences in the tweet. This features provide a measure of the length of the tweet (number of words, sentences, and total lemmata) as well as its lexical complexity (number of unique lemmata).

### 3.5. Feature Selection

Feature selection and dimensionality reduction are key parts in text classification problems, as having too many features might hamper generalization and could result in overfitting [62]. There are many methods for selecting relevant features, usually divided into three main categories: (i) Wrapper methods, such as the *Las Vegas Wrapper* [63]; (ii) filter methods, such as *Chi-square* [64]; and (iii) embedded methods, such as *LASSO* (Least Absolute Shrinkage and Selection Operator) [65], which is a regression analysis method that performs variable selection and, to a lesser extent, regularization to enhance the prediction accuracy and interpretability of the statistical model it produces.

In this paper, feature selection is applied only when using frequency-based features and is carried out using a filter method based on feature sparsity and variance, combined with a *LASSO* model.

### 3.6. Document Classification

Supervised classification consists of predicting the value of a qualitative response variable. This response variable is commonly known as category or class. There are many different classifiers, and their performance depends on the problem. We refer the reader to the paper by Jordan and Mitchell [66] for an overview on the state-of-the-art of classifiers in machine and statistical learning. As there is not a classifier that is the best for all kinds of problems (*no free lunch theorem*, Wolpert and Macready [67]), for *HaterNet*’s design, a set of models have been evaluated:

- **Logistic regression** (LR) uses features(predictors) for building a linear model that estimates the probability that an observation belongs to a class. It is possible to apply feature selection or shrinkage by introducing L1 (lasso) or L2 (ridge) penalizations, respectively.
- **Random Forests** (RF): Ensemble classifiers whose construction is based on the use of several *decision trees* [68]. Each decision tree is trained with a sample bootstrapped from the training dataset and using a random subset of the features. Decision trees partition the factor space according to value tests, therefore resulting in a nonlinear classification. The nodes of the trees are determined so as to maximize the information gain. There are different criteria for determining this information gain being Gini and entropy two of the most common.
- **Support Vector Machines** (SVM) are classifiers whose result is based on a decision boundary generated by support vectors, i.e., the points closest to the decision boundary. The shape of the boundary is determined by a kernel function. In this way, it is possible to solve problems that cannot be solved by a linear boundary. Intuitively, a good separation is achieved by the hyperplane that has the largest distance to the support vectors, as, in general, the larger the margin the lower the generalization error of the classifier.
- **Linear Discriminant Analysis** (LDA) is a classifier based on Bayes’ theorem, which requires modeling the distribution function of continuous features. Classification is made by using Bayes’ rule to compute the posterior probability of each class, given the vector of the observed attribute values. Bayes rule assumes that the features are conditionally independent given the category.
- **Quadratic Discriminant Analysis** (QDA), as LDA, is a classifier based on Bayes’ theorem. However, *QDA* assumes that each class has its own covariance matrix.
- **Neural Networks** are one of the most popular classifiers currently used in ML and crime prediction [69]. There are two important types of *neural networks*: (i) *Feedforward Networks* (*FFN*), that have no loops and (ii) *Recurrent Neural Networks* (*RNN*), which both process sequences of data and take into account the instant of time that each piece of data is processed. Therefore, they are more useful for solving NLP problems. In these types of network, the output of the neurons is not only based on the input values, but also on the previous outputs. For this reason, *RNN* are generally trained using *back-propagation through time* (*BPTT*) [70].

#### Classification Metrics

**Figure 2:**
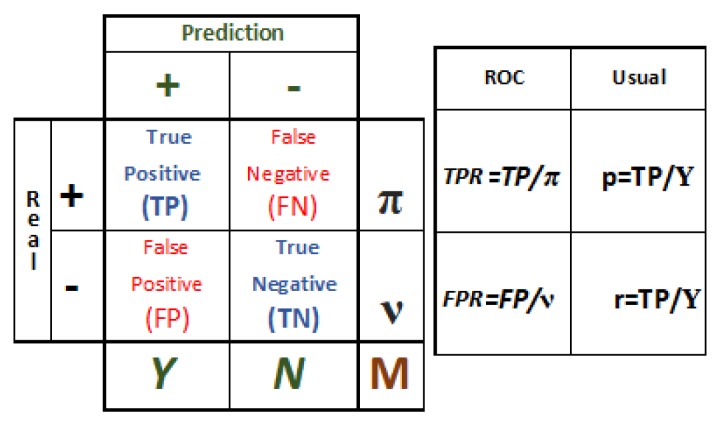
Confusion matrix and classification metrics terminology.

The above classifiers have been evaluated in our system according to the following metrics [71] (see Figure 2 for references to the symbols in the formulas).

1. ***Accuracy*** measures the overall performance of the classifier.
  $$\mathrm{acc}=\frac{TP+TN}{TP+FP+TN+FN}$$
  Unfortunately, accuracy is not a significant performance measure when the dataset is imbalanced. Thus, other metrics, such as the following, should be considered.
2. ***Precision*** (also called positive predictive value) is the fraction of relevant instances among the retrieved instances:
  $$p=\frac{TP}{TP+FP}$$
3. ***Recall*** (also called sensitivity or true positive rate) is the fraction of relevant instances that have been retrieved over the total amount of relevant instances:
  $$r=\frac{TP}{TP+FN}$$
4. ***F1 score*** is the harmonic mean of precision and recall:
  $$F1=2\cdot \frac{p\cdot r}{p+r}$$
5. ***False positive rate*** is the fraction of nonrelevant instances that are consider relevant over the total amount of relevant instances:
  $$\mathrm{FPR}=\frac{FP}{TN+FP}$$
6. **ROC curve and Area under the ROC curve** (also called *AUC* or *AUCROC*). The ROC curve is obtained by plotting the true positive rate (TPR) against the false positive rate (FPR). The AUC is the area under the resulting ROC curve.

## 4. Hate Speech Detection: Implementation

Having reviewed the main stages and theoretical concepts used in our system, *HaterNet*’s specific implementation details and configuration are next illustrated. Note that the steps described in Section 4.2 and Section 4.3 are only necessary for labeling the initial corpus on which *HaterNet*’s supervised learning methods are trained.

### 4.1. Tweet Collection and Cleaning

The first task is gathering a corpus that will be the basis for the development and implementation processes. For this first version of *HaterNet*, a corpus composed of tweets collected at different random dates between February 2017 and December 2017—having a final size of 2M tweets—has been created. This strategy of collecting tweets at different dates is an approach for reducing the problem of the *triggering events* mentioned by Downs [2], as more topics and events are captured than in shorter observation periods. The tweets are collected using the Twitter Rest API [72]. In each request, a *json* file is received with the tweets’ content and metadata, including the UTC offset, which is used to identify the tweets originating from Spain. Once *HaterNet* was trained and running only the latest tweets written in Spanish are retrieved for their evaluation and monitoring. The rationale is to obtain the most recent tweets from Spain written in Spanish.

#### Data Cleaning

As mentioned in Section 3, *HaterNet*’s cleaning task consist of preparing the downloaded raw text for processing. A tweet’s content is coded in Unicode format, so it may contain: emojis, tildes, Chinese, and other non-ASCII characters. To avoid codification problems, all non-alphanumeric characters are removed and the text is converted to lower case and tokenized. Also, during this stage, symbols that do not add semantic content are deleted while new expression tokens are created to unify “colloquial” expressions that refer to the same concepts (e.g., laugh and surprise). Examples of generated tokens are shown in Table 2.

This process is done through the design of rules and regular expressions that identify both the tokenized content and the normal text. For example, to detect if there is a hashtag in a tweet, the following regular expression is used; “\#[0-9a-zA-z]”, which means that it detects as a hashtag everything that begins with # and is followed by alphanumeric characters. Here, the hashtag is tokenized as *HASHTAG*, whereas the text that follows is kept following a designed rule. As previously mentioned, this process is also used to create general tokens meant for unifying words/concepts that can be expressed/represented in different ways as well as to detect possible misspellings. For example, “jaaja”, “jaja”, and “jajaj” in spanish mean the same, and therefore are classified in the same token, i.e., TOKENLAUGH.

Note that in this cleaning process emojis have been preserved and tokenized as they add semantics to the analyzed tweets. Examples of emojis and their codes can be found in Table 3.

**Figure 3:**
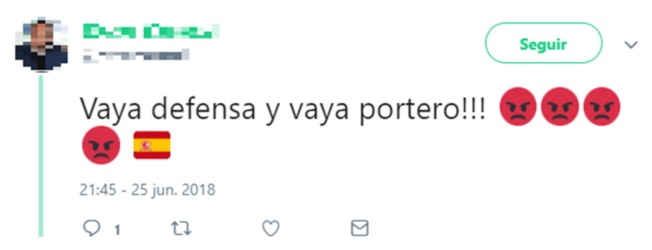
The importance of emojis. Literal meaning of the text: “What a defense and what a goalkeeper!!!”

The tweet represented in Figure 3 shows the importance of emojis to obtain semantic information.

In this case, the red face emoji shows that the user that sent the tweet is angry (without this information the textual content is not enough to ascertain the polarity of the message), and the flag shows that the defense and goalkeeper the tweet is refereeing to belongs to the Spanish national soccer team. Note that different variants of the same *emoji* are detected, as exemplified by the two bottom emojis in the Table 3. In our case, the *Emoji*’s skin color can be very valuable for determining if the hate speech in a tweet is about race or ethnicity.

### 4.2. Tweet Selection

**Figure 4:**
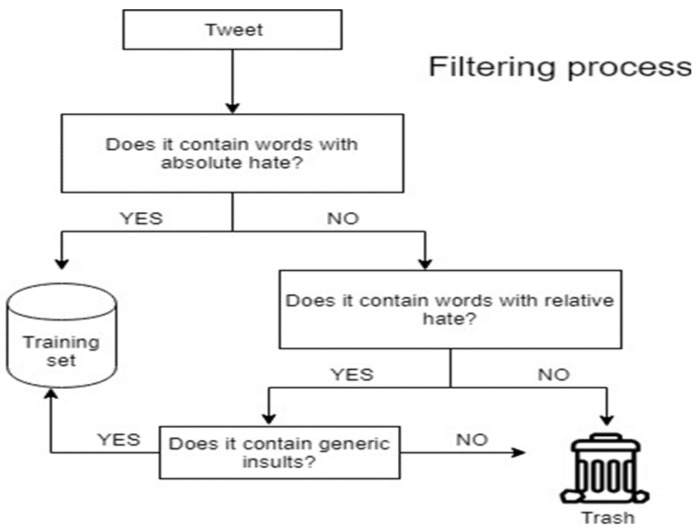
Filtering process.

As motivated in Section 3.2, it is necessary to apply a filter to the corpus of two million tweets prior the manual labeling process. The filter (sketched in Figure 4) is designed as a two-step process that makes use of seven files. Six of them are dictionaries of words that represent different types of hate speech (i.e., ethnicity, race, gender, disability, politics, and religion), whereas the last one contains generic insults.

**Figure 5:**
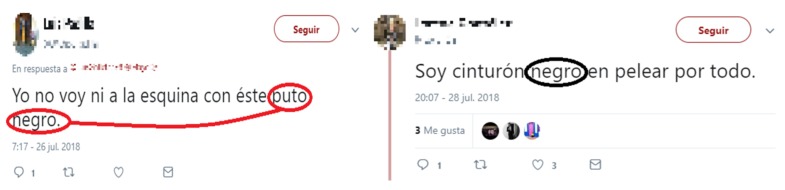
Hate and non-hate tweets. Literal translation and meaning: (i) “I won’t even go to the corner with that f***ing negro” (meaning: I will not have anything to do with that black person); (ii) 11I’m a black belt in fighting for everything” (meaning: I never give up).

Words inside each of the first six files are tagged with two possible degrees of hate: absolute or relative. A word is said to contain absolute hate if the word unequivocally expresses hate, regardless of its context. Otherwise, if hate depends on the context, the word is said to contain relative hate. For example: “feminazi” contains absolute hate, whereas “negro”, which also denotes the color black in Spanish, may contain hate or not depending on the context. At this point, each tweet is represented as a vector of elements. Therefore, for this process, each element in the vector is scanned using the first six files of the filter. If the tweet contains at least one element tagged as absolute hate, then it is selected as possible container of hate speech and passes the filter. If, on the other hand, it contains an element tagged as relative hate, then the context of that term is assessed using the seventh file of the filter: if at least one of the elements of the tweet appears in this file, then the tweet is selected as possible container of hate speech and, therefore, passes the filter. For example, if the word “negro” (tagged as relative hate) appears in a tweet, the rest of the terms are scanned in the second step of the filter. If the tweet contains a generic insult, then the tweet is likely to contain hate and is included in the new reduced corpus to be labeled. The example in Figure 5 shows two real tweets where the generic insult vocabulary is important to ascertain their meaning.

The filter described has been designed to focus explicitly on the type of hate speech considered in this study (see Section 1). Therefore, the bias introduced by the generic insult vocabulary ensures that the content of the tweet might be civilly or criminally punishable.

Note that, after applying the filter, only 8710 tweets out of the original 2 m in the raw corpus were selected for labeling.

### 4.3. Tweet Labeling

At this stage, all tweets that passed the filter were labeled by hand. To label the corpus, the *SNOAHC-SES* selected four experts with different backgrounds:

- Labeler A: 44-year-old, public servant.
- Labeler B: 23-year-old, Psychology graduate.
- Labeler C: 24-year-old, Law graduate.
- Labeler D: 23-year-old, Criminology graduate.

Each expert read the whole filtered corpus and indicated for each tweet if they considered it contained hate speech or not according to the definition given (see Section 1). The final label of each tweet has been decided by majority vote. As there is an even number of labelers, it is possible to incur in a tie. In this event, a fifth person (Labeler E, a 49-year-old professor of Computer Science) casts the deciding vote. For an inter-agreement analysis of the labelers, please refer to Section 5.

At the end of this stage, due to time restrictions, 6000 tweets out of the 8710 filtered were labeled. Using the majority vote method, 1567 tweets (26% of the corpus) have been labeled as hate containers, whereas 4433 (74% of the corpus) have been labeled as non-hate containers. Both the initial 2M tweets dataset and the labeled dataset are available at DOI: 10.5281/zenodo.2592149.

### 4.4. Tweet Representation

At this point an NLP preprocessing pipeline is followed: lemmatization, stopword removal, and POS tagging. Therefore, at the end of this stage, each tweet is represented as a vector of unigrams, i.e., words, emojis, POs tags, or tokens.

With respect to suffixes detection, we have developed an *ad hoc* algorithm, as there is no library for detecting suffixes in Spanish. In particular, a word contains a suffix if it ends with one of the suffixes contained in the last appendix of the Dictionary of the Royal Spanish Academy [73]. Identified suffixes are also added to the vector tweet representation.

Next, as explained in Section 3.4, each tweet is converted into one of these two representations, depending on the model: frequency-based and embedding-based.

#### 4.4.1. Frequency-Based Representation

When using the frequency-based representation, each tweet *i* is represented by a vector $v_{i}$ that includes four frequencies (i.e., absolute, binary, logarithm, and ratio) for each unigram considered (i.e., words, POS tags, emojis, suffixes, and expression tokens), plus four more elements for the number of words, lemmata (total and unique), and sentences in the tweet. Using the labeled corpus, 9586 different unigrams (containing words, tokens, or emojis), 56 different POS tags, and 149 different suffixes were found. That is, each text is annotated by (9586+56+149)×4+4=39,168 different features.

#### 4.4.2. Embeddings-Based Representation

This second representation is based on natural language semantics, where the semantic of each term in the tweets is obtained using a word2vec model. Unigram embeddings are obtained using the *Gensim Python* library for NLP. The neural network for the embeddings is trained using the unfiltered and unlabeled 2M tweets corpus. This allows taking advantage of all the semantic information of the whole corpus, which poses an improvement, as the method recognizes more vocabulary. This comes especially in handy when *HaterNet* analyzes new tweets which include tokens that are not present in the labeled corpus. In this case, a classification model estimated on the embedding-based representation would be able to take advantage of this information, while a frequency-based model would not.

**Figure 6:**
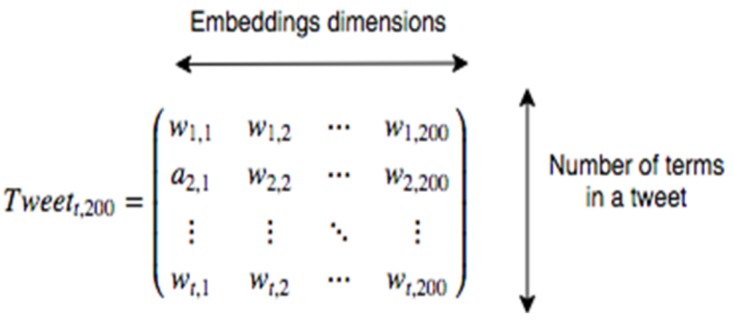
Matrix representation of a tweet. *t* is the number of unigrams in the tweet.

The neural network used for building the embeddings for words, emojis and expression tokens has 200 neurons in the hidden layer, this means that the generated embeddings have 200 dimensions. Therefore, when using this configuration each tweet is represented by a matrix having as many rows as unigrams and 200 columns (see Figure 6).

These embeddings can be extended by attaching them the corresponding suffixes, POS tags, and tf-idf information. When such information is not available, a vector of zeros of the appropriate size is attached instead. In particular, suffixes embeddings are trained separately using word2vec with 10 neurons, POS tags are represented using one-hot encoding, and tf-idf is a single real value.

### 4.5. Feature Selection

The step of feature selection is applied only when using the frequency-based representation. Initially, a filter is applied on the absolute frequencies to remove vector elements with high sparsity (i.e., less that 1% non-zeroes) or very low sparsity and variability (i.e., more than 99% non-zeroes and very low coefficient of variation). This allows disregarding vector elements that could only be used to discriminate a tiny fraction of the documents and that, therefore, could lead to overfitting. After this filter, only 1.65% of the initial vector elements are preserved. After applying this method, the original vocabulary (having a size of 9791) is reduced to 245 elements. For these elements, the four studied frequency measures (i.e., absolute frequency, binary, logarithm, and ratio transformations) are computed. After this first filter, the feature space dimension is 245×4+4=984.

Next, a *LASSO* algorithm using a *logistic regression model* with *L1* penalization is applied. Leave-one-out cross-validation is used to choose the best penalization parameter and, consequently, the final set of features. After this step, the number of selected features is 148. This reduced space dimension is reasonable, as tweets are short messages, and is more suitable for the classification stage.

When using the embedding-based representation it is not necessary to apply feature selection as (i) it is not logical to delete embeddings (columns in the matrix), as they contain different semantics of a tweet’s unigrams (rows in the matrix), and (ii) 200 features are not considered excessive for the classification stage.

### 4.6. Classification

Due to their differences, the frequency-based and the embedding-based representations require the use of different classification models, which are presented in the following.

#### 4.6.1. Frequency-Based Representation

The features obtained after the selection stage are used to train the following supervised classification models; *LDA*, *QDA*, *Random Forest*, *Ridge Logistic Regression*, and *SVM*.

#### 4.6.2. Embeddings-Based Representation

**Figure 7:**
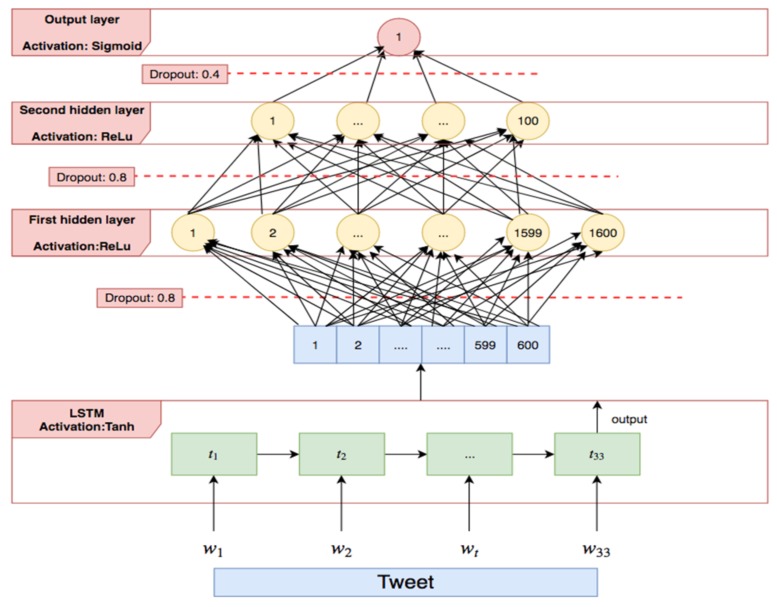
*LSTM*+*MLP* architecture.

As tweets in this iteration are represented in a matrix form, it is not possible to directly apply classifiers, as the number of rows in the matrix is variable and depends on the number of unigrams in the tweets. However, there are approaches that allow to transform this matrix representation into a single vector [74]. For example, a tweet could be represented as the tf-idf weighted sum of all the word embeddings that it contains. Unfortunately, this simple methodology does not take into consideration the order of the words, which is extremely relevant as it affects the meaning of a sentence, e.g., “poor unfortunate men” and “unfortunate poor men”. Thus, it is necessary to look for a way to take into account the words’ order. The solution implemented is a recurrent neural network, concretely a *LSTM neural network*, described by Graves [75]. Before training the recurrent neural network, there needs to be a fixed number of terms. In our case, this is the maximum number of terms found after preprocessing the tweets of the labeled corpus, which is 33. All tweets that contain less than 33 terms have the remaining rows filled with 0 s (padding technique). The designed neural network architecture is shown in Figure 7.

The *LSTM* is combined with a multilayer perceptron (MLP) with two hidden layers. Word embeddings are processed by the *LSTM* neural network in each time step, one at a time, preserving word order. The output of the *LSTM* neural network is processed by an hyperbolic tangent activation function. This output is a vector of dimensions 600. The MLP network consists of the input layer (600 neurons), two hidden layers (1600 and 100 neurons with ReLu activation function), and the output layer (a single neuron with sigmoid activation function). Dropout units have been placed between each pair of layers, having associated probabilities of 0.8, 0.8, and 0.4, respectively. The dropout units assign to each input neuron a probability of elimination during training and they are used to avoid overfitting.

If the value provided by the output neuron is above a threshold, then the classifier considers that the tweet contains hate speech; otherwise, the neural network considers that there is no hate content in the tweet.

#### 4.6.3. Parameter Estimation

The classification models considered have a number of parameters that need to be estimated in order to achieve optimal classifications. For this purpose, different values of these parameters are considered and the performance of the resulting models is evaluated.

In this work, the *Area Under the Curve* (*AUC*) (Section 3.6) is used for determining the goodness of the classifiers, as we are facing an imbalanced classification problem and are interested in the general performance of the classifier for different probability thresholds.

**Figure 8:**
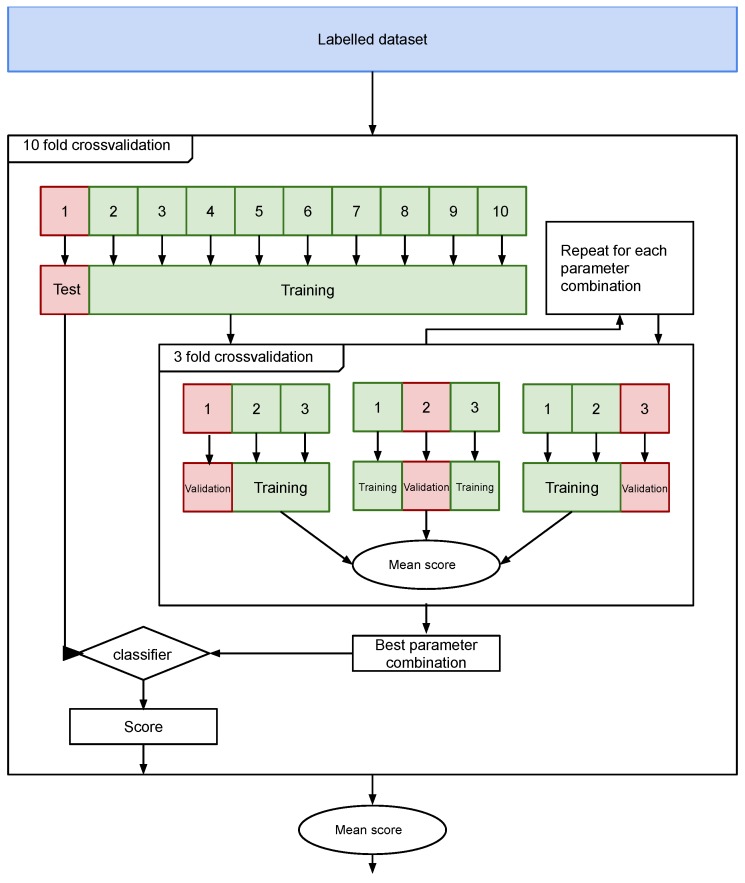
Classifiers’ performance estimation and hyperparameter tuning diagram.

The performance of the classifiers is estimated using 10-fold cross-validation. Hyperparameter tuning is based on a grid search using a 3-fold cross-validation. A grid search allows to find instances of classifiers with all the combinations of specified by the user. This stage has a high computational cost. Figure 8 shows a diagram of the validation procedure.

## 5. Results

This section presents the results of the experiments and a discussion of their significance. First, the inter-rater agreement among the labelers is calculated. Then, the representativeness of the embeddings is evaluated. Finally, the classification models considered are evaluated and compared to identify the best, which is chosen to be included in *HaterNet*. In the following section, the performance of *HaterNet* is compared to that of the models from the literature.

### 5.1. Labelers’ Inter-Rater Agreement

When using human evaluators, it is important to measure their degree of agreement. The two main statistical measures of the agreement percentage, popular in labeling processes and areas such as psychology and education [76], are *Cohens’ kappa* [77], which measures the degree of agreement between two raters, and *Fliess’ kappa* [78], which makes it possible to measure the concordance among two or more raters. Both measures are considered to be more robust than the simple agreement percentage. For both measures, the agreement κ is defined as

(1) $$\kappa =\frac{p_{o}-p_{e}}{1-p_{e}}=1-\frac{1-p_{o}}{1-p_{e}}$$

where $p_{o}$ represents the relative observed agreement and $p_{e}$ measures the hypothetical chance agreement probability. κ=1 means that the degree of agreement is maximum among users, κ=0 indicates that the degree of agreement is obtained just by pure chance, and κ≤0 means that the concordance is worse than rating randomly.

The average inter-agreement among the labelers is κ=0.588(σ=0.081), denoting moderate agreement according to the qualitative interpretation proposed by Landis and Koch [79].

### 5.2. Word Embeddings Representativeness

The goodness of the generated word embeddings has been checked using a quantitative and a qualitative approach: (i) *Accuracy*, where 91% of the times the word2vec trained neural network (detailed in Section 4.4.2) predicts correctly the neighbors of a word in the corpus. (ii) *Random Sampling*, where some words were taken from the vocabulary and five of their nearest neighbors were analyzed. Cosine similarity is used for calculating how close two word embeddings are

$$Cos\left(W_{1},W_{2}\right)=\frac{W_{1}\cdot W_{2}}{\left\|W_{1}\left\|\right\|W_{2}\right\|}$$

where $W_{1}$ and $W_{2}$ represent the two word embeddings.

Table 4 (first column) shows some words (translated from Spanish) taken from the corpus and five of their nearest neighbors. It can be seen that columns are related to the term’s meaning, i.e, colors, body parts, months, mobile phones, food, and soccer players.

As far as emojis are concerned, they have been decoded and included in the tweet corpus and their embeddings seem to correctly capture their semantics (see Table 5).

As shown in the table, some emojis are included among the nearest neighbors of some terms from the corpus and vice versa. The last row in the table illustrates that, also among the emojis, semantics has been captured: for example, an angry face emoji’s meaning is close to that of a red angry face. After analyzing the results presented in this section, we can conclude that there are grounds to state that the obtained word embeddings satisfactorily capture the semantics.

### 5.3. Classifiers’ Performance Results

This section analyzes the performance of the classification models considered. Table 6 shows the results of our experiments. As illustrated in the table, different combinations of features, classification models, and thresholds have been considered. Overall, 17 different models have been tested. For each of them, the precision, recall, F1, and AUC have been computed using cross-validation on the best parameters found, as explained in Section 4.6. It is important to underline that models 1–5 have the same structure as the one proposed by Quijano-Sánchez et al. [23], and they have been included for comparison purposes. We consider that the most significant performance measure is the AUC, as it is not affected by the threshold chosen. Therefore, the best models are 7, 13, and 9 that achieve an AUC of 0.828. However, model 7 uses less features, therefore, following the principle of parsimony, it is chosen as the best model and selected for production in *HaterNet*. Model 7 enriches token embeddings with td-idf and classifies tweets using LSTM+MLP.

Determining the best threshold is still relevant to the problem at hand, as *HaterNet* has to provide a definitive classification to the final user. The *SNOAHC-SES* has specified that their greater need is to analyze a small number of tweets containing hate speech. Thus, the probability threshold has been set to 0.7, as it obtains a *precision* of 0.784. This comes at a cost, as the *recall* is rather low (0.333). However, due to the high number of tweets available, this is not a major problem.

The results obtained allow to draw some interesting insights. Concerning the features considered, the following conclusions can be drawn. According to Table 6, including suffixes and POS tags does not improve the classification for the embedding-based models, as the AUC does not change (AUC = 0.828). However, the results for the frequency-based models are mixed. Most models perform slightly worse when including emojis and suffixes. The only classification model that benefits from their inclusion is the QDA. However, its AUC is still lower than those of all the other models. We can conclude that suffixes and POS tags do not have a significant impact on the classification. On the other hand, td-idf improves the performance of the embedding-based models. Finally, in the context considered, embedding-based models perform better than the frequency-based.

## 6. Comparison of *HaterNet* against State-Of-The-Art Approaches and Discussion

As illustrated in Table 6, the best model obtained is 7, which achieves a better AUC than models 1–5. This means that, for the data considered, the double neural deep learning approach proposed in this paper improves on the methodology presented by Quijano-Sánchez et al. [23]. This, in turn, means that our model outperforms all the models that Quijano-Sánchez et al. [23] compared against the authors of [46,47,48,49,50,51].

To further assess the relevance of the methodology proposed, we have applied model 7 to a number of datasets from the literature and compared the performance of *HaterNet* against those reported. Concretely, Araque et al. [43] used several Twitter datasets and proved the improvement in the performance of their ensemble approaches against well-know state-of-the-art approaches, specifically the approaches followed by Go et al. [80], Manning et al. [81], Narayanan et al. [82], Smedt and Daelemans [83], Loria [84], and Kathuria [85]. Out of all the datasets used by Araque et al. [43], only *Vader* [86] and *STS-Gold* [87] are fully reproducible. The best F1 score obtained by the authors were 0.895 and 0.892 for the *Vader* and *STS-Gold* datasets, respectively, (note that we provide the F1 measure as it is the one given in the revised paper). Table 7 shows *HaterNet*’s performance on the aforementioned datasets where, again, our model wins the comparison.

Analyzing both our reported results and those in the state-of-the-art of hate speech (see Table 1), a number of interesting multidimensional aspects can be concluded:

- Regarding the employed datasets, most approaches use the same datasets to compare themselves against [5,6,8,9,10,11]. These datasets were originally proposed in [5], where authors manually annotated 16k tweets labeled as “sexist”, “racist”, or “clean”, and in [6], where authors designed a new dataset of 6k tweets (3k being part of the previous dataset), using both expert and amateur annotators. These datasets, along with the one presented in [7], are, to the best of our knowledge, the only publicly available hate speech datasets. Unfortunately, it is no longer possible to use the first two datasets as benchmarks as the authors provided only the ids of the tweets to be downloaded; as also reported in [8], Twitter has deleted several of them, mainly due to their offensive content. For instance, out of Waseem and Hovy [5] original 16k tweets, only 11k are available. Therefore, it is not possible to compare to the results reported using these datasets. This reality stresses the importance of one of this paper’s contributions, that is, the necessity of providing open datasets for reproducibility and benchmarking.
- Regarding the inter-annotator agreement, only the datasets described in [5,56] reported their κ coefficient being 0.57 and 0.26, respectively. Again the lack of details in the related literature hinders more profound and transversal analysis. However, it allows us to (i) conclude that our reported parameter of κ=0.588 falls within normal boundaries and (ii) agree with the conclusion reached in Waseem [6], Del Vigna et al. [56], and Ross et al. [55], that the annotation of hate speech is a hard task.
- Regarding the studied features, the models achieving the best results on the datasets in the literature are [9,11]. All of them make use of embeddings: [9] combines character and word embeddings, whereas [11] uses random word embeddings. Also, *HaterNet* relies on embeddings; specifically, on word, emoji, and expression embeddings. Differently from the previous models, in *HaterNet*, the embeddings are enriched by adding the tf-idf which, as explained in Section 5.3, helps the classification and improves the performance of the embedding-based models. This analysis suggests that, in the context of hate speech detection in Twitter, embedding-based methods outperform frequency-based models.
- Regarding the implemented classification approaches, different classical machine learning models have been studied throughout the years, with LR, NB, DT, RF, and SVM being the most common  Most studies have so far reported that SVM outperforms the others; this is the case in [7,53,57]. However, as pointed out by [7], LR has the advantage of allowing a more transparent and comprehensible interpretation of the results, being the observed performance sometimes even better or not significantly different. Our results reported in Table 6 and [23] support this affirmation.
  Also, a significant group of researchers have applied neural network-based approaches to implement classifiers that detect hate speech in social media content [8,9,10,11,56]. When comparing this approaches to other machine learning methods, the performance of NN clearly outperforms the latter; this conclusion is supported by this paper’s results and those of the related literature [10].
- Regarding this paper’s novelty and contribution with respect to other hate classifiers, as previously mentioned, not having public datasets makes it difficult to benchmark. Besides, the lack of details given in the papers in the literature also makes it difficult to reproduce their results; this is, for example, in the case of [9]. As previously reported in Section 2.4, only three of the reviewed papers provide the source code or enough implementation details). However, the approaches presented in [5,6,7,52,53,54,57] make use of either LR, SVM, NB, or RF methods. These have been shown, both in [10,11,56] and in this research, to be inferior when compared to NN methodologies. Therefore, we can conclude that this paper’s model would essentially outperform all of them.
  With regards to the papers that implement a NN approach, all of them, except for [56], test their approaches on common previously published datasets. However, it is currently not possible to obtain all the tweets comprising the datasets as some of them have been removed from Twitter, as previously explained. Due to the impossibility of testing our best model on these datasets, a fair comparison could be obtained by testing the best models in the literature [9,11] on our dataset. However, as mentioned earlier, [9] does not provide neither the source code nor sufficient details for the reimplementation of their methodology. Therefore, we could test on our dataset only the combination of LSTM with Random Embedding and GBDT by Badjatiya et al. [11]. The results are illustrated in Table 8.
  The model by Badjatiya et al. [11] obtains an AUC of 0.788, which is inferior to the AUC obtained by our best model, 0.828. Therefore, in the context of our data, model 7 is preferable to the model by Badjatiya et al. [11].
  The main difference between these models is that, in the case of Badjatiya et al. [11], the word embeddings are generated using only the labeled tweets; whereas, in the present case, we use the full dataset of 2M tweets. A second significant discrepancy is that Badjatiya et al. [11] generate document embeddings by averaging the word embedding, which could result in a significant loss of information. Finally, we include emojis and tokens embeddings and enrich all the embeddings with additional tf-idf information. These differences in the implementation could cause the gap in terms of performance and should be further investigated in future research.
  All in all, to the best of our knowledge, it can be concluded that our double deep learning approach, which uses token embeddings enriched with the tf-idf, outperforms the best models from the literature on text classification.

## 7. Social Network Analyzer

So far, we have presented the details relative to the tasks of collecting and classifying tweets. However, as mentioned in Section 1, *HaterNet* has another key application: the analysis and monitoring of hate in Twitter. In this section, we introduce the second module of our system: the *Social Network Analyzer*. Social Network Analysis uses graph theory techniques to investigate social structures [88] and offers innovative insights by targeting information more effectively [89]. The literature presents many researches that combine social media, sentiment analysis, and graph analysis metrics (using likes, hashtags, etc.) to perform trend analysis [90,91]. However, to best of the authors knowledge, there is no single approach that enables finding the distribution of communities by using concurrency of words in tweets, or the targeted source of the topic. As stated in the introduction, our goal is to provide a visual thermometer of emotions that allows to map the hate state of a territory and, therefore, to monitor its evolution and to take measures by targeting concepts, emitters, and receivers of hate.

The high number of tweets containing hate speech identified by *HaterNet* makes it impossible to analyze their content manually to extract information patterns and trends. Therefore, it is desirable for the final user to have an expert system that automatizes and supports this task. To this end, the *Social Network Analyzer* has three main functionalities that will be next detailed: (i) word cloud tab, (ii) users’ mentions tab, and (iii) terms tab. These visualization tools use as input the following information:

- The Hate Speech Detection module output, i.e., the set of tweets classified as hate speech containers and the associated probability.
- The most common terms in the selected tweets, their frequency, and a list of the document indexes where they appear. This ranking only includes adjectives, nouns, and emojis.
- Word embeddings reduced to two dimensions using a *t-distributed stochastic neighbor embedding* (t-SNE) technique, which is a dimensional reduction technique for maintaining relative distances between words in the new space [92].
- A directed graph built on user’s mentions. In the graph, nodes represent users and an arc (A,B) is created when user A mentions B in a tweet.
- A non-directed words concurrency graph based on document appearance. In the graph, the nodes represent words and two nodes are connected by an edge if the corresponding words appear in the same tweet.

### 7.1. Word Cloud Tab

**Figure 9:**
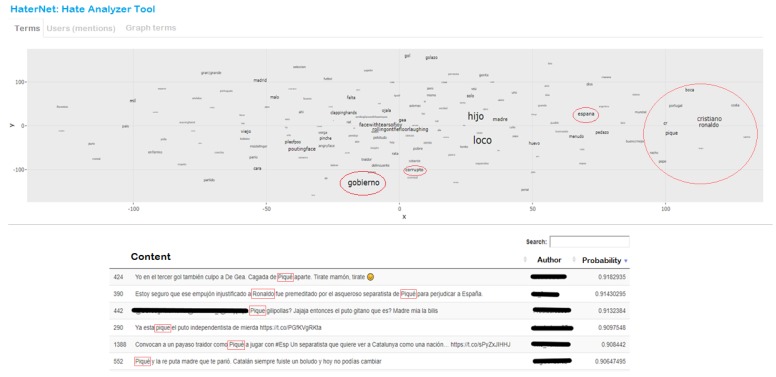
Example of main tab. Users’ names have been censored for privacy reasons.

The main interface of the application is dedicated to analyzing terms, as shown in Figure 9.

The main tab shows a semantic word cloud of the most frequent adjectives and nouns found in the tweets that contain hate. Usually, word clouds are plots of terms whose size varies proportionally to the terms’ frequency, i.e., terms’ size increase as frequency does. Besides, as in this project we have used word embeddings, we can take advantage of this fact to plot terms which are semantically related closer to each other. However, before plotting these terms, it is necessary to use dimensional reduction techniques. In this case, t-SNE is used.

The user can interact with the graph by zooming in and out to better visualize the different hate terms and by passing the mouse over the terms and observe their frequency.

The upper part of Figure 9 shows an example of a semantic word cloud in *HaterNet* where the tweets with hate content were captured the day of the Spain vs. Portugal soccer match of the 2018 World Cup. This is reflected in the graph with soccer players located very close to each other and with the word “Cristiano Ronaldo” standing out as a very common term. The second tab, located below the semantic word cloud, contains a table with all the hate tweets that were captured. This table allows the user to sort the rows by column values. In this case, the user can sort the table by author’s name or by the probability of containing hate. This helps police officers to focus on the tweets with high probability of containing hate. The designed interface allows the user to interact with data tables. Therefore, it is possible for the user to click on a term to show the tweets that contain that term in the data table. Figure 9 shows an example of a user that clicked on “Pique” in the graph and sorted the data table by the probability of containing hate. Being able to identify relevant nouns and adjectives inside the identified hate messages provides useful applications: (i) It allows for the comparison of hate trends and to study its evolution by looking at the cloud of words at different periods of time; this, in turn, can result in actions taken to prevent/mitigate specific hate waves or in the study of possible triggers or origins (e.g., another highlighted noun reflected in Figure 9 is the government itself). (ii) It serves as an explicative tool, allowing to visualize and analyze the terms that the classifier identifies inside hate messages, especially those highlighted by being bigger than the rest. This feature can be used to detect possible biases. (iii) It allows the user (in this case, the police) to cluster messages by topic/terms and analyze common patterns or triggers. (iv) It allows to further cluster topics/terms (and their corresponding messages) related to each other. As a consequence, analysts can associate and study similar hate aspects together, which permits the detection of common patterns that can be used for surveillance or for designing preventive measures. These last two actions are further supported by the functionality of the terms’ tab, described in Section 7.3.

### 7.2. Users’ Mentions Tab

The main purpose of this second tab is to analyze the way hate messages propagate in Twitter. Usually, when a user mentions another user in a tweet that contains hate speech, it is because the first one is insulting the second one or because the second one is related to topics that the first one does not like. These relations can be represented in a graph where a user (node) is connected to another if the first one is the author of the tweet in which the second one is mentioned.

**Figure 10:**
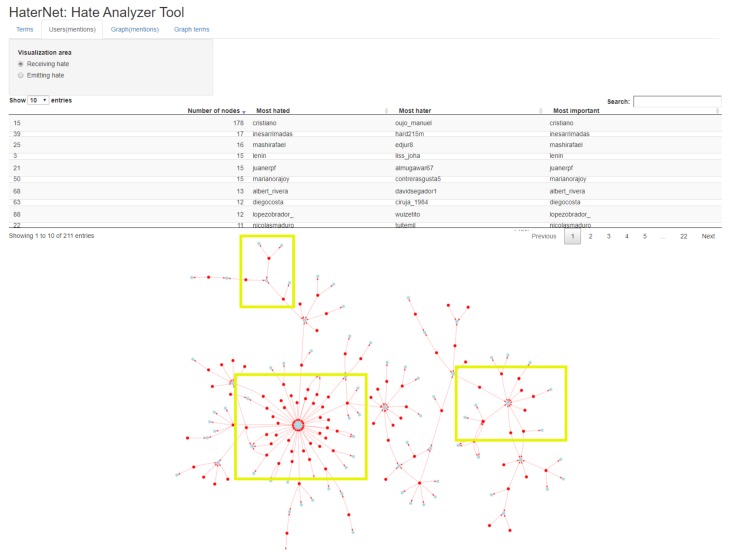
Example of the users’ mentions tab.

This tab summarizes information in two ways: (i) As a table (see Figure 10 top), where we can see the number of nodes in the component, the highest in-degree in the graph, the highest out-degree, and the *PageRank* [93]. The node with the highest in-degree could be interpreted as the most hated, whereas the node with the highest out-degree is the node that emits hate the most. *PageRank* is used to measure the importance of each node in the network in terms of hate received. (ii) As a graph (see Figure 10 bottom), where a red node represents a user that has sent hate content through Twitter, whereas a blue one is related to a user that received messages with hate content.

The current tab also includes a check box in order to adapt the nodes’ size proportionally to their in or out degree. By doing so, if the graph is too large, the user can check which nodes to filter. It also allows the user to drag a node, zoom in/out, and highlight the neighborhood of a node. Extra functionalities have been added to ease the task of hate analysts, e.g., when passing the mouse over an edge the tweet that originated the drawn relation appears.

This functionality is novel to hate speech detectors. In fact, previous works [12] have focused on the message and not on the actors involved. As shown in Figure 10, drawing the actors as a connected network of related people that emit or receive hate allows to easily visualize and highlight, which users are especially being the focus of hate messages (see the big blue node in the right part of the graph) or which ones are acting as generators of hate (see the red nodes surrounding it). It also allows to detect peculiar relationships (e.g., two nodes mutually hating each other in the bottom left part of the graph), or particularly “agressive” nodes (e.g., the red, centered star in the top left part of the graph, which represents a user hating seven others). This, in turn, can trigger different policing measures related to both protection and surveillance.

### 7.3. Terms’ Tab

The last tab is dedicated to visualizing the word concurrency graph. Edges are weighted by the frequency two terms appear together in the same tweet. Next, the *Louvain* community detection algorithm is applied [94]. The objective is to allow analysts to be able to discover relationships between events by identifying clusters (painted with different colors) and isolating them for further study.

**Figure 11:**
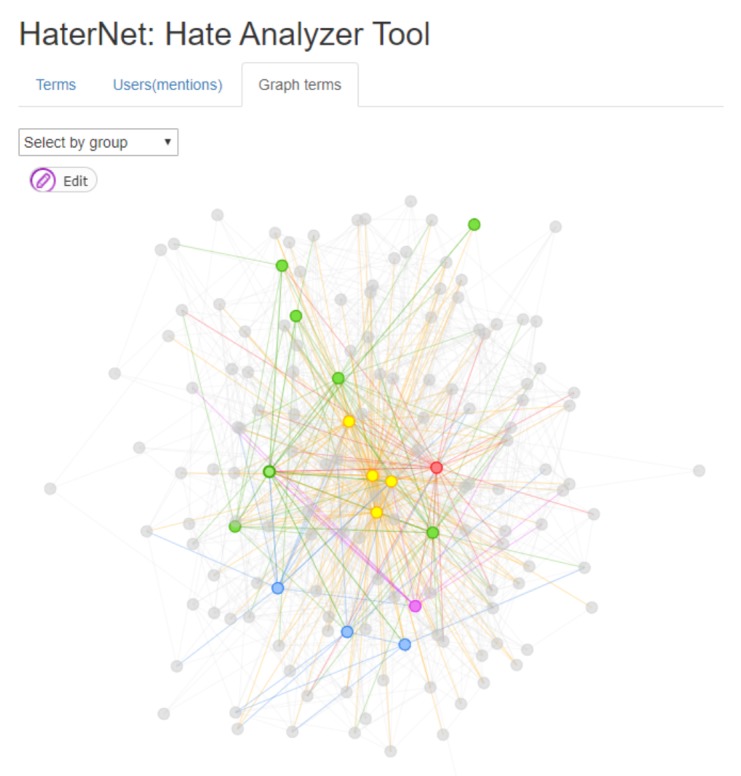
Terms neighborhood highlighting.

Extra functionalities have been added to allow analysts to isolate communities, remove nodes, or highlight a node’s neighbors (see Figure 11).

### 7.4. Applications

**Figure 12:**
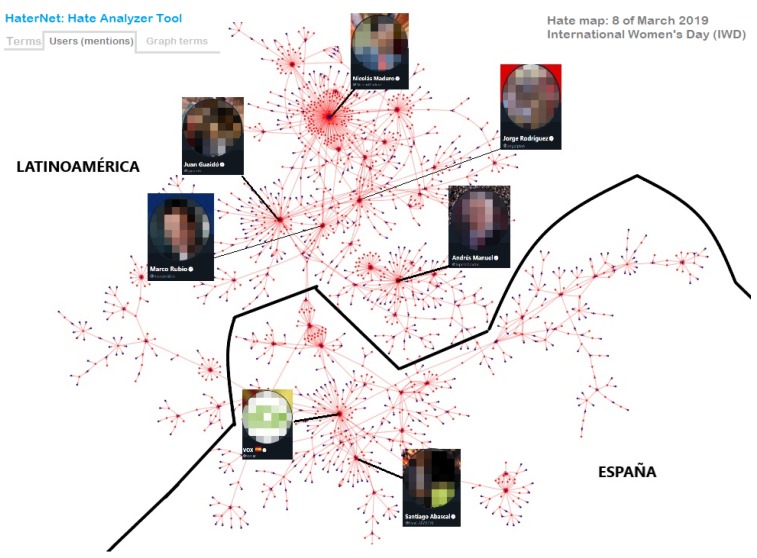
Twitter’s hate map in Spain on the 8 March 2019 (International Women’s Day) was focused on an extreme right political party and its leader.

*HaterNet* is already in use as an observatory of phenomena for significant events and days, such as the International Women’s Day, the gay pride parade, or soccer matches (see Figure 12 for an example). The *SNOAHC-SES* has been studying for months the results of both modules: (i) the lists of tweets identified as hate containers of the *Hate Speech Detection* module, and (ii) the evolution of the hate cloud and the different tabs of the *Social Network Analyzer* module. Concrete examples of hate trends discovered are out of the scope of this paper. However, we here highlight some of the activities that the use of this system has led to:

- Analysis of tweets tagged by *HaterNet* as hate speech containers including their symbology (e.g., emojis).
- Analysis and classification of “tweeter” communities that share messages with toxic content, as well as the permanence and evolution of hate speech in networks produced by a relevant social events.
- Statistics on relevant events, words and terms, used as a support tool for the police units with Twitter “Trusted Flagger” licenses, for the elimination of hate content.

## 8. Implemented Architecture

**Figure 13:**
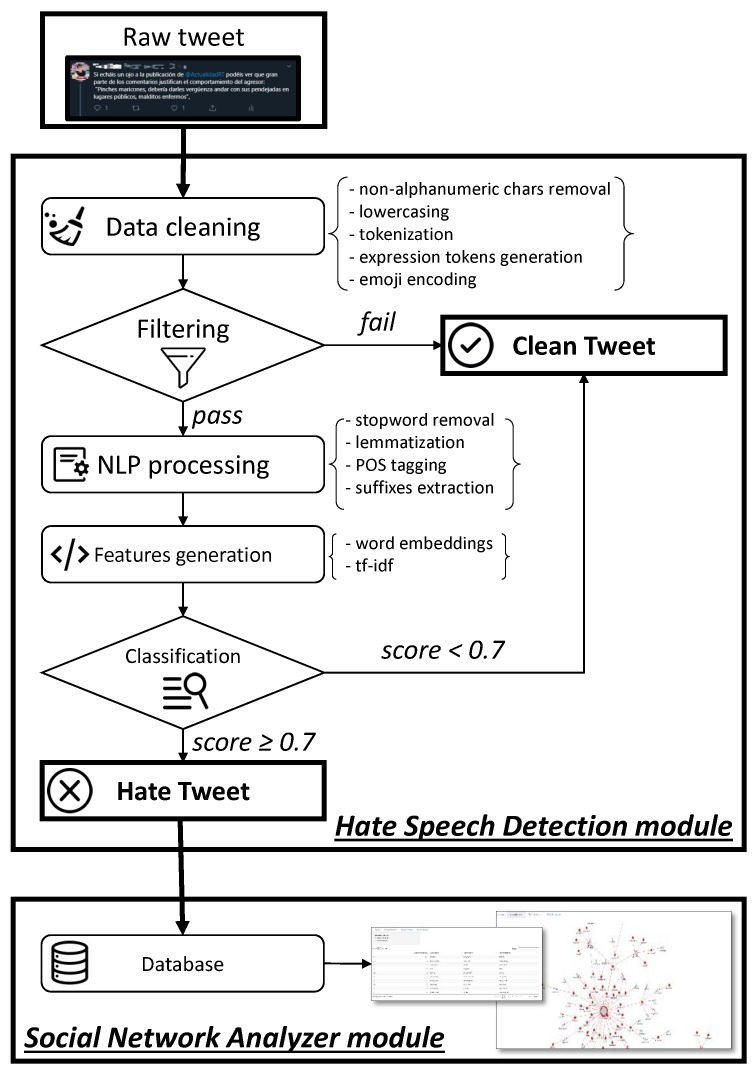
Implemented architecture.

This section summarizes the functioning of *HaterNet* as a whole. To better illustrate the process, Figure 13 graphically illustrates all the different steps.

When a new tweet is obtained for analysis, first it has to be processed by the Hate Speech Detection module (Section 3) that classifies it as hate or clean.

Inside of the module, the tweet undertakes a number of steps, detailed in the following. The first step is data cleaning (Section 4.1), which standardizes the content of the tweet. Next, the cleaned tweet is passed through a rule-based filter (Section 4.2) that ascertains whether the tweet might contain hate speech or not. If the tweet fails the test, then it is classified as clean and it is not further processed. Otherwise, it has to be analyzed by the classification model. Therefore, the tweet needs to be prepared for the task, by undertaking an NLP processing step (Section 4.4), and then its features are extracted (Section 4.4). The classifier model assigns a score that represents the probability that the tweet contains hate speech (Section 4.6). If this score is lower than the threshold, 0.7, then the tweet is classified as clean; otherwise, it is classified as hate by the module.

Once the Hate Speech Detection module terminates its analysis, if the tweet contains hate, then it is passed to the Social Network Analyzer module (Section 7) that stores the tweet in a database. The tweets in the database are then processed by the module which represents the information obtained in the word cloud, users’ mentions, and in the terms tabs (Section 7.1, Section 7.2, and Section 7.3, respectively).

## 9. Conclusions

This paper presents *HaterNet*, an intelligent system for the detection and analysis of hate speech in Twitter. *HaterNet* has been developed in collaboration with the *Spanish National Office Against Hate Crimes*, and it is currently in use to monitor the evolution of hate in Social Media. It is comprised of a novel text classification model to detect hate speech and a social network analysis module to monitor and visualize its state and evolution.

The design of the text classification module has been experiment-driven. We have compared 19 different strategies, each consisting of a combination of features and classification models. Finally, the best model, which achieves the highest AUC of 0.828, uses embeddings of words, emojis, and token expressions, and then enriches them by including tf-idf. Other features, such as POS tags and suffixes, have been tested and it has been found that they have no positive impact. The classifier implemented in *HaterNet* is a double deep learning model, which combines an LSTM and an MLP neural network and frequency features. Our approach has been compared with previous models from the literature and it has been found that *HaterNet* outperforms all of them.

Moreover, the designed visualization tool is the first of its kind to provide a user-friendly representation of those terms that appear in tweets containing hate speech and, also, of the interaction between users in tweets. This module organizes information in a simpler and more visual way through interactive tables and graphs that allow the study of users’ relationships, communities, and, overall, makes it easier to monitor the state of hate in Twitter.

Looking at the practical implications of this research to the *Spanish National Office Against Hate Crimes*, *HaterNet* represents an advancement in terms of analysis capabilities—manually evaluating the amount of daily generated tweets is unaffordable—and application possibilities. As mentioned in the previous section, monitoring and studying the identified tweets can help establish an early warning that allows to take predictive actions against potential hate crimes. Besides, *HaterNet* has recently been used to analyze hate in Twitter during Spanish elections [95] and to study its impact on LGTBI communities [96].

Future possibilities for the extension of *HaterNet* and research opportunities are highlighted in the following.

- Classification of tweets according to the type of hate expressed, e.g., racist, homophobic, and xenophobic. This classification could be used as an open source of information by organizations, observatories, or specialized NGOs.
- Adapting the system to other domains aimed at police investigation, e.g., terrorism, gender violence, or cyberbullying.
- Strengthening knowledge and capacities of institutions and civil society by having an online hate speech thermometer.
- Establishing an early warning alert that allows to take action against the potential impact of hate speech.
- Understanding the correlation between hate speech and crimes with hate motivation finally reported to the police. This would allow testing of the hypothesis that hate speech is the prelude to hate crime.
- Automatically removing toxic content from SM, or penalizing its appearance in the rankings. Also, *HaterNet* could be used to identify possible criminal content, as a previous step to safeguarding this information, and then pursuit of possible legal prosecution.

We hope that this work will be a useful source of ideas for future research on text classification and will contribute further in the development of more sophisticated and accurate models for the detection of hate speech. 

## Figures and Tables

**Table 1:** Summary of the main characteristics of hate detection models in the literature. The first two columns show the paper and the year of publishing. Next, the features considered (in bold the features used in the best model), the classification models tested (best one in bold), the datasets used in the experiments, and the availability of the used dataset (an asterisk (*) indicate that only a fraction of the original dataset is available) are reported. Finally, the performance of the best model found is illustrated: accuracy, precision, recall, F1 score, and AUC. A dash (-) indicates that the value is not available.

Paper	Year	Features	Model	Dataset	Available	Accuracy	Precision	Recall	F1 Score	AUC
Djuric et al. [52]	2015	BOW, TF, TF-IDF, **paragraph2vec embeddings**	**LR**	951,736 Yahoo Finance user comments	No	-	-	-	-	0.8007
Zia et al. [53]	2016	**unigrams, TF-IDF, retweets, favourites, page autenticity**	**SVM**, NB, kNN	tweets	No	-	0.971	0.97	0.971	-
Silva et al. [54]	2016	**sentence structure**	**rule based**	27.55M whispers and 512M tweets, unlabeled. 100 labeled messages.	No	-	1	-	-	-
Waseem and Hovy [5]	2016	**Author gender**, length of tweets, length of user description, location, **char n-grams**, word n-grams	**LR**	16,914 annotated tweets	Yes *	-	0.7293	0.7774	0.7393	-
Waseem [6]	2016	**char n-grams**, **word n-grams**, **skip-grams**, **tweet length**, author gender, **clusters**, **POS**, Author Historical Salient Terms (AHST)	**LR**	6909 annotated tweets	Yes *	-	0.9250	0.9249	0.9119	-
Badjatiya et al. [11]	2017	char n-grams, TF-IDF, BoWV, **random embeddings**, GloVe embeddings	LR, RF, SVM, **GBDT**, DNN, CNN, **LTSM**	[5]	Yes *	-	0.930	0.930	0.930	-
Davidson et al. [7]	2017	n-grams, TF-IDF, POS, readability, sentiment, hashtags, mentions, retweets, URLs, length	**LR**, NB, DT, RF, SVM	24,802 labeled tweets	Yes	-	0.91	0.90	0.90	-
Gambäck and Sikdar [8]	2017	**word2vec embeddings**, random embeddings, char n-grams	**CNN**	6655 tweets from [6]	Yes	-	0.8566	0.7214	0.7829	-
Park and Fung [9]	2017	**char embeddings**, **word embeddings**	CharCNN, WordCNN, and **HybridCNN**	[5,6]	Yes *	-	0.827	0.827	0.827	-
Del Vigna et al. [56]	2017	POS, sentiment analysis, **word2vec embeddings**, CBOW, n-grams, text features, **word polarity**	SVM, **LSTM**	6502 annotated Facebook comments	No	0.7523	0.732	0.7371	0.731	-
Salminen et al. [57]	2018	**n-grams**, **semantic and syntactic**, **TF-IDF**, **word2vec embeddings**, **doc2vec embeddings**	LR, DT, RF, Adabost, **SVM**	5143 labeled comments YouTube and Facebook videos	No	-	-	-	0.96	-
Zhang et al. [10]	2018	n-grams, POS, TF-IDF, mentions, hastags, length, readability, sentiment, mispellings, emojis, punctuation, capitalisation, **word embeddings**	SVM, **CNN + GRU**	[5,6,7] and 2435 annotated tweets	Yes * [5]; Yes * [6]; Yes [7]; No [10]	-	-	-	0.82 in [5]; 0.92 in [6]; 0.82 in [5,6]; 0.94 in [7]; 0.92 in [10]	-

**Table 2:** Example of tokens created to unify expressions.

Semantic	Token Type
URL	TOKENURL
Mention	USER
Hashtag	HASHTAG
Question mark	TOKENQUES
Exclamation mark	TOKEXC
Laughing face: XD	TOKENXD
Quotation marks	TOKENCOMI
Laughs: jaja, ajaj, jajaj	TOKENLAUGH
Surprise: WTF, wtf	TOKENWTF

**Table 3:** Sample emojis.

Emoji	Code
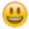	:smiling_face_with_open_mouth:
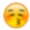	:kissing_face_with_closed_eyes:
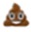	:pile_of_poo:
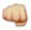	:oncoming_fist::light_skin_tone:
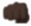	:oncoming_fist::dark_skin_tone:

**Table 4:** Word embeddings’ nearest neighbors (translated from Spanish).

Term\Nearest n.	1st	2nd	3rd	4th	5th
red	yellow	green	blue	orange	white
head	back	leg	neck	belly	stomach
january	march	september	february	june	august
samsung	galaxy	xiaomi	lg	snapdragon	huawei
food	dinner	supper	snack	eat	breakfast
messi	neymar	ney	cristiano	umtiti	mathieu

**Table 5:** Emojis embeddings semantics (translated from Spanish).

Reference Embedding	Nearest Neighbor
smacker	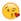
sandwich	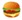
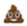	shit
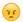	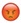

**Table 6:** Performance statistics for the models considered: precision, recall, F1, and AUC.

Model ID	Features Type	Features Considered	Classification Model	Classification Threshold	Precision	Recall	F1	AUC
#1	Frequency based	Unigrams, POS tags	Ridge R.	0.5	0.655	0.382	0.483	0.798
#2			SVM		0.685	0.329	0.445	0.79
#3			RF		0.809	0.167	0.277	0.769
#4			QDA		0.341	0.789	0.476	0.651
#5			LDA		0.641	0.42	0.507	0.796
#6	Embeddings based	Words, emojis, and expression tokens	LSTM+MLP		0.62	0.572	0.595	0.823
#7		Words, emojis, expression tokens, and tf-idf			0.625	0.598	0.611	**0.828**
#8	Frequency based	Unigrams, POS tags, suffixes, emojis, expression tokens	Ridge R.		0.639	0.417	0.505	0.794
#9			SVM		0.629	0.392	0.483	0.777
#10			RF		0.746	0.165	0.27	0.766
#11			QDA		0.386	0.71	0.5	0.71
#12			LDA		0.622	0.422	0.503	0.79
#13	Embeddings based	Words, emojis, expression tokens, suffixes, POS tags, tf-idf	LSTM+MLP		0.625	0.598	0.611	**0.828**
#14	Frequency based	Unigrams, POS tags, suffixes, emojis, expression tokens	Ridge R.	0.7	0.794	0.219	0.343	0.794
#15			SVM		0.786	0.19	0.306	0.777
#16			RF		1	0.005	0.001	0.766
#17			QDA		0.382	0.72	0.495	0.71
#18			LDA		0.741	0.272	0.398	0.079
#19	Embeddings based	Words, emojis, expression tokens, suffixes, POS tags, tf-idf	LSTM+MLP		0.784	0.333	0.467	**0.828**

**Table 7:** Performance statistics of Model 7 on datasets from the literature: precision, recall, F1, AUC.

Dataset	Precision	Recall	F1	AUC
*Vader*	0.923	0.890	0.906	0.925
*STS-Gold*	0.898	0.888	0.893	0.914

**Table 8:** Performance statistics of the best model proposed by Badjatiya et al. [11] (LSTM+Random Embedding + GBDT) on our dataset: precision, recall, F1, AUC.

Threshold	Precision	Recall	F1	AUC
0.7	0.787	0.123	0.212	0.782
0.5	0.647	0.394	0.490	0.782

## Abbreviations

The following abbreviations are used in this manuscript:

SM	Social Media
SES	Security of the Ministry of Interior
SNOAHC-SES	Spanish National Office Against Hate Crimes
NLP	Natural Language Processing
ML	Machine Learning
DNN	deep neural network
KDE	Kernel Density Estimation
BOW	Bag Of Words
POS	Part-of-Speech tagging
AUC	Area Under the Curve
SVM	Support Vector Machines
CNN	Convolutional Neural Network
RF	Random Forests
DT	Decision Trees
LDA	Linear Discriminant Analysis
QDA	Quadratic Discriminant Analysis
FNN	Feedforward Networks
RNN	Recurrent Neural Networks
BPTT	back-propagation through time
GDBT	Gradient Boosted Decision Trees
MLP	Multilayer Perceptron
LSTM	Long Short-Term Memory
GRU	GRU Gated Recurrent Unit Networks
LR	Logistic Regression
